# The role of aryl hydrocarbon receptor in vitiligo: a review

**DOI:** 10.3389/fimmu.2024.1291556

**Published:** 2024-02-01

**Authors:** Yiting Li, Yibin Zeng, Zile Chen, Xi Tan, Xingyu Mei, Zhouwei Wu

**Affiliations:** ^1^ Department of Dermatology, Shanghai General Hospital, Shanghai Jiao Tong University School of Medicine, Shanghai, China; ^2^ Department of Dermatology, Minhang Hospital, Fudan University, Shanghai, China

**Keywords:** aryl hydrocarbon receptor, vitiligo, oxidative stress, immunity modulation, melanogenesis

## Abstract

Vitiligo is an acquired autoimmune dermatosis characterized by patchy skin depigmentation, causing significant psychological distress to the patients. Genetic susceptibility, environmental triggers, oxidative stress, and autoimmunity contribute to melanocyte destruction in vitiligo. Due to the diversity and complexity of pathogenesis, the combination of inhibiting melanocyte destruction and stimulating melanogenesis gives the best results in treating vitiligo. The aryl hydrocarbon receptor (AhR) is a ligand-activated transcription factor that can regulate the expression of various downstream genes and play roles in cell differentiation, immune response, and physiological homeostasis maintenance. Recent studies suggested that AhR signaling pathway was downregulated in vitiligo. Activation of AhR pathway helps to activate antioxidant pathways, inhibit abnormal immunity response, and upregulate the melanogenesis gene, thereby protecting melanocytes from oxidative stress damage, controlling disease progression, and promoting lesion repigmentation. Here, we review the relevant literature and summarize the possible roles of the AhR signaling pathway in vitiligo pathogenesis and treatment, to further understand the links between the AhR and vitiligo, and provide new potential therapeutic strategies.

## 1 Introduction

Vitiligo is a common depigmentation disease characterized by white macules and patches with distinct margins (1). It was reported that approximately 0.5-2% of the population worldwide is affected, and more than half of the patients develop the disease between the ages of 10 and 30 years (2). Although genetic susceptibility, environmental triggers, oxidative stress, autoimmunity, and neural hypothesis are recognized as the pathogenic factors (3), the pathogenesis of vitiligo is incompletely clarified and there is no satisfactory therapy available right now.

Aryl hydrocarbon receptor (AhR) is a nuclear transcription factor activated by exogenous and endogenous ligands. In the canonical pathway, cytoplasmic AhR binds ligands and translocates into the nucleus, subsequently combining with the aryl hydrocarbon receptor nuclear translocator (ARNT). The AhR/ARNT complex recognizes xenobiotic regulative elements (XREs) and mediates numerous toxicological or biological effects by modulating the transcription of various downstream genes (4). Besides, AhR can also regulate the gene expression through non-canonical signaling pathways, such as nuclear factor-κB (NF- κB), Krüppel-like factor 6 (KLF6), and the estrogen receptor (ESR) (5–7). Accumulating evidence has evinced that AhR is highly expressed in the skin and plays important roles in regulating epidermal barrier differentiation, cellular homeostasis, pigment synthesis, and skin immunity (8–10). In recent years, the role of AhR in the pathogenesis of various skin diseases such as non-melanoma skin cancer, melanoma, psoriasis, atopic dermatitis, acne, and hidradenitis suppurativa has been reported (11–16). The AhR agonist tapinarof has been used to treat psoriasis and atopic dermatitis (17–20).

Recent studies identified abnormal AhR expression in vitiligo (21–23). Rekik et al. found decreased AhR transcription in the lesional skin of vitiligo (21). Wang et al. showed that decreased AhR expression in peripheral blood mononuclear cells of vitiligo patients was closely associated with the disease severity (22). In addition, the relation between AhR gene functional mutations and vitiligo susceptibility has been suggested (23). Current opinions reveal that AhR signaling pathway is essential for vitiligo treatment through antioxidant, immune modulation, and melanogenesis effects (24–26). Activation of AhR signaling conduces to progression control and repigmentation, showing great potential in vitiligo treatment. Here, we review the relevant literature, summarize the possible roles of AhR pathway in vitiligo pathogenesis, and conclude the current AhR agonists and antagonists studied in vitiligo, to advance our knowledge and provide new insight into potential therapy.

## 2 AhR protects melanocytes from oxidative stress damage

Oxidative stress is recognized as the critical triggering factor in melanocyte death and the initial event of autoimmune response in vitiligo (27). The excessive reactive oxygen species (ROS) induced by exogenous or endogenous stimuli cause DNA damage, mitochondrial dysfunction, lipid peroxidation, and endoplasmic reticulum stress, ultimately leading to melanocyte death and autoantigen production which can activate adaptive immunity and enhance the attacks on melanocytes (28, 29).

Nuclear factor erythroid 2-related factor 2 (Nrf2) is a transcription factor and makes a significant contribution to redox homeostasis (30). When the Nrf2 is activated, the Keap1-Nrf2 complex breaks down, and the released Nrf2 binds to antioxidant-response elements (AREs), transcribing and upregulating the expression of several antioxidant enzymes (31). Recent opinions suggest that the Nrf2-ARE signal transduction is disordered in vitiligo melanocytes, causing antioxidant incapacity and melanocyte damage (32). Aspirin, simvastatin, and ginsenoside Rk1 can activate Nrf2 and protect melanocytes against oxidative stress damage, suggesting the Nrf2-ARE pathway could be a significant target for vitiligo therapy (33–35).

**Figure 1:**
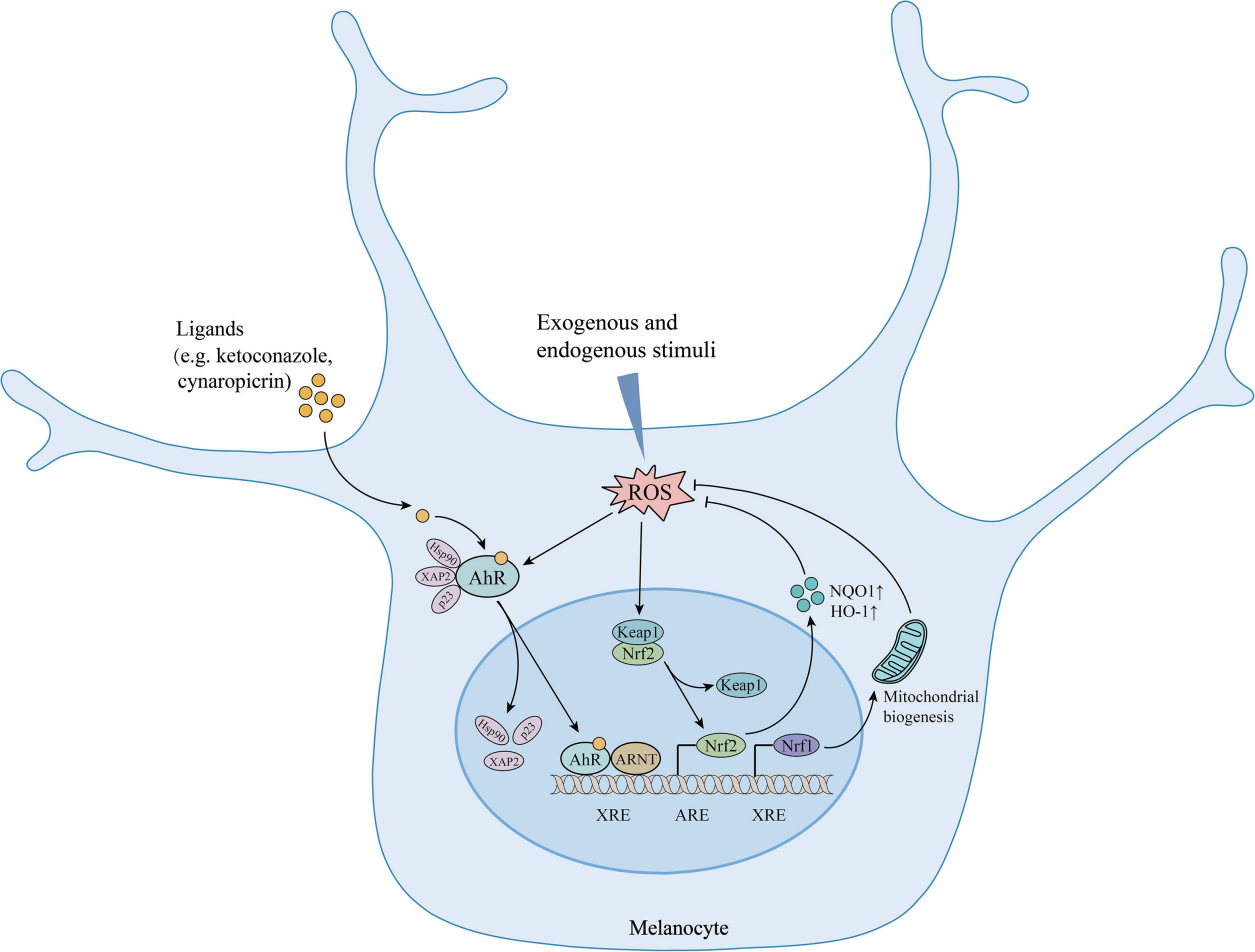
The protective effects of AhR signaling in melanocytes under oxidative stress. AhR activation upregulates Nrf2/ARE pathway and increases the expression of antioxidative enzymes. Meanwhile, AhR upregulates Nrf1 expression and promotes mitochondrial biogenesis, ultimately reducing oxidative stress-induced mitochondria and melanocyte destruction. AhR, aryl hydrocarbon receptor; Hsp90, heat shock protein 90; XAP2, hepatitis B virus X-associated protein 2; p23: co-chaperone protein; ARNT, aryl hydrocarbon receptor nuclear translocator; XRE, xenobiotic regulative element; ROS, reactive oxygen species; Nrf2, nuclear factor erythroid 2-related factor 2; Keap1, Kelch-like ECH-associated protein 1; ARE, antioxidant response element; HO-1, heme oxygenase-1; NQO1, NAD(P)H:quinone oxidoreductase 1; Nrf1, nuclear respiratory factor 1; ↑, upregulation.

AhR has been recognized as a master switch for antioxidation. It has been reported that the inhibition of the AhR pathway enhanced H2O2-induced oxidative stress damage and apoptosis in melanocytes (36). It has also been verified that AhR signaling pathway activation can protect cells from oxidative damage via Nrf2-dependent antioxidant system (30, 37). Nrf2 can be either directly activated by ROS or other pathways such as AhR. In fact, Nrf2 has been identified as one of the target genes of AhR. AhR activation directly modulates the Nrf2 pathway and upregulates the expression of antioxidative enzymes, such as heme oxygenase-1 (HO-1) and NAD(P)H:quinone oxidoreductase 1 (NQO1), which are key molecules in maintaining the redox homeostasis of melanocytes (38). Whether AhR activation could trigger the Nrf2 pathway may depend on the ligand and cell type. The ligand, such as ketoconazole, has been proven to differentially activate the AhR/Nrf2 pathway, which inhibited BaP-induced ROS in keratinocytes (39). Besides, AhR signaling pathway is also involved in repairing mitochondrial oxidative damage, which plays a key role in mediating melanocyte apoptosis and death. AhR can upregulate Nrf1 and its downstream TFAM expression to promote mitochondrial biogenesis and avoid oxidative damage in mitochondria and melanocytes (36). In addition, AhR signaling probably crosstalk with PI3K/Akt and Wnt/β-catenin pathway as well, which are related to reducing oxidative stress-induced melanocyte destruction (35, 40, 41). AhR can upregulate the pathways of PI3K/Akt and Wnt/β-catenin, and activate their downstream signal Nrf2, thereby reducing oxidative stress (**Figure 1**).

## 3 AhR signaling is involved in autoimmune response regulation

**Figure 2:**
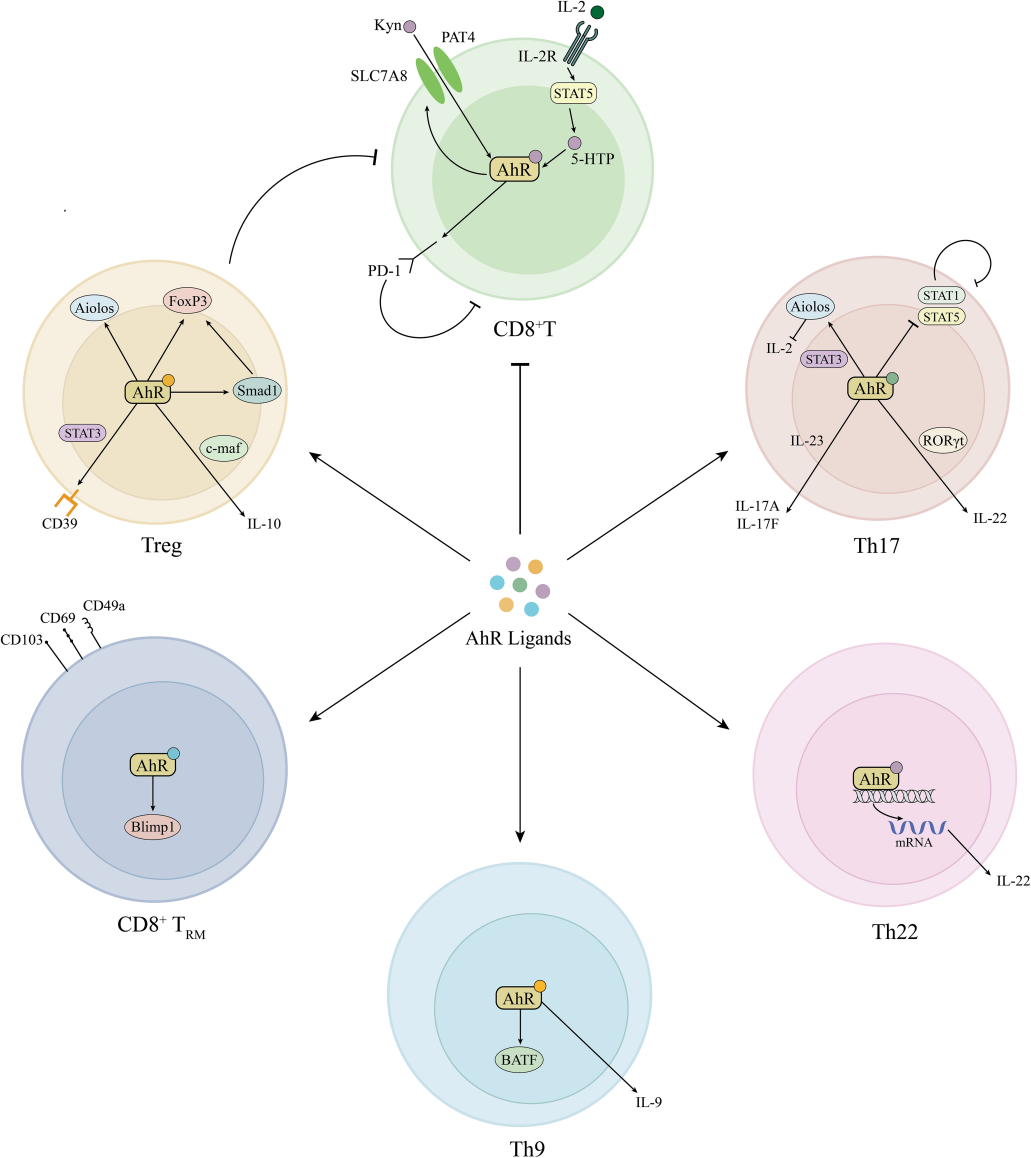
Roles of AhR signaling in adaptive immune modulation. AhR induces PD-1 expression in CD8^+^T cells by the activation of Kyn-AhR and STAT5-5-HTP-AhR pathways and simultaneously promotes Treg cell differentiation, together inhibiting abnormal CD8^+^T cell activation in vitiligo. In addition, AhR is also involved in the differentiation of Th9, Th17, Th22, and T_RM_ cells. Kyn, kynurenine; AhR, aryl hydrocarbon receptor; PD-1, programmed cell death protein-1; STAT, signal transducer and activator of transcription; 5-HTP, 5-hydroxytryptophan; IL, interleukin; FoxP3, forkhead box P3; Smad1, decapentaplegic homolog 1; c-maf, musculoaponeurotic fibrosarcoma; CD39, cluster of differentiation 39; Treg, regulatory T; RORγt, RAR-related orphan receptor γt; Th, T helper; Blimp1, B lymphocyte induced maturation protein 1; BATF, Basic Leucine Zipper ATF-Like Transcription Factor.

The involvement of autoimmunity is crucial in the development of vitiligo (42, 43). Endogenous and exogenous stress signals stimulate melanocytes to release heat shock protein 70 (HSP70). HSP70 stimulates the secretion of IFN-α by dendritic cells, which induces keratinocytes to produce chemokines CXCL9 and CXCL10 and recruits CD8^+^T cells expressing CXCR3 receptor, ultimately leading to the targeted autoimmune destruction of melanocytes (44). Recently, multiple cytokine subsets along with regulatory T (Treg) cell imbalance were found in vitiligo. Czarnowicki et al. found that the levels of T-helper (Th) 1, Th2, Th9, Th17, and Th22 cells were significantly increased in the peripheral blood of vitiligo patients, along with Treg cell counts decreased, suggesting multidimensional immune disorders in vitiligo (45). AhR signaling plays important roles in modulating T cell differentiation and function, which is likely to be an appealing target to combat abnormal autoimmune in vitiligo (46) (**Figure 2**).

### 3.1 AhR inhibits the function of CD8^+^ T cells

CD8^+^ T cells are vital to melanocyte destruction and disease progression. It is well known that IFN-γ-CXCL9/10-CXCR3-CD8^+^ T cell axis is crucial in the destruction of melanocytes (47, 48). CD8^+^ T cells secrete IFN-γ, activating the Janus kinase (JAK) 1/2 dimer and inducing the production of CXCL9 and CXCL10 from surrounding keratinocytes to further recruit additional T cells to the epidermis through a positive-feedback loop (48). Targeting these molecules in this pathway, including IFN-γ, the IFN-γ receptor, JAK1 and JAK2, signal transducer and activator of transcription (STAT)1, CXCL10, and CXCR3, may be appealing therapy in vitiligo (49, 50). Increasing evidence has shown that oral and topical JAK inhibitors are efficacious and safe for vitiligo and other dermatosis (51–54). The JAK1/2 inhibitor ruxolitinib cream has been approved by FDA for treating non-segmental vitiligo patients over 12 years old (55). Other emerging JAK inhibitors, including ritlecitinib and baricitinib, are under the clinical trial stage (53, 56). Besides, it has been proved that CD8^+^ T cells could mediate melanocyte apoptosis by secreting perforin or granzymes and Fas-FasL mechanisms (57, 58).

AhR plays an important role in the regulation of CD8^+^ T cell responses (59). Lawrence et al. showed that TCDD-mediated AhR activation indirectly inhibited the primary CD8^+^ T cell response to the influenza virus by modulating the dendritic cell function (60). Similarly, CD8^+^ T cells exposed to AhR agonist TCDD during development showed a reduced response to influenza virus infection in later life (61). In addition, Liu et al. demonstrated that kynurenine (Kyn) could activate AhR and upregulate the expression of PD-1 as well as SLC7A8 and PAT4. The latter two molecules are the main Kyn transporters in CD8^+^ T cells, thereby forming a positive feedback loop and leading to CD8^+^ T cell inhibition (62). Furthermore, they also found that IL-2 could activate STAT5-5-HTP-AhR axis and induce tumor-reactive CD8^+^T cell exhaustion (63). All the evidence indicates that AhR could probably inhibit the function of CD8^+^ T cells, thus regulating abnormal autoimmunity. Besides, recent literature has shown the crosstalk between AhR and JAK-STAT pathway. Furue showed that IL-13/IL-4‒JAK‒STAT6/STAT3 axis inhibited the transcription of FLG, LOR, and IVL mediated by AhR (64). Cai et al. revealed that Benvitimod could activate AhR and inhibit the JAK/STAT3 pathway in keratinocytes (65).

### 3.2 AhR promotes the differentiation of Treg cells

Treg cells facilitate immune homeostasis by inhibiting immune activation and maintaining peripheral self‐tolerance (66). It has been reported that Treg cells suppressed autoreactive CD8^+^ T cells by inhibiting proliferation and cytokine secretion (67). Accumulating evidence has shown that decreased numbers and suppressive function of Treg cells resulted in an unrestricted autoimmune response and melanocyte damage in vitiligo (68–70). Chen et al. found the conversion of normal Tregs to Th1-like Tregs in the peripheral blood and lesional skin of vitiligo patients indicating the impaired suppression on CD8^+^ T cell proliferation and activation (71). Replenishing peripheral skin Treg cells was proved to interfere with depigmentation and normalize off-balance immune responses (72, 73).

AhR activation can mediate the differentiation of Treg cells by various mechanisms including transcription regulation and epigenetic modification (74–76). To be concrete, AhR induces CD39 and IL-10 in Treg cells, as well as leading to epigenetic changes in the forkhead box P3 (FoxP3) locus and upregulating the expression of Aiolos and Smad 1, which control the expression of FoxP3 and repress IL-2 transcription, and ultimately promoting Treg cell differentiation and combating autoreactive CD8^+^ T cells.

### 3.3 AhR regulates the differentiation of Th17 cells

Previous research suggested that elevated Th17 cells and IL-17 levels might be correlated to the duration, extent, and activity of vitiligo (77). Il-17 produces the chemokine CCL20, attracting CD8^+^ T cells and killing the melanocytes (77, 78). In addition, IL-17 stimulates the endothelial expression of E- and P-selectins as well as the adhesion molecules ICAM-1 and VCAM-1, then enhancing the migration of neutrophils, which results in ROS production and subsequent autophagic cell apoptosis in vitiligo (77, 79, 80). Inhibition of IL-17 expression has been proved to improve vitiligo lesions (81).

AhR is highly expressed in Th17 cells and promotes the early differentiation of Th17 cells through multiple mechanisms (82, 83). AhR cooperates with STAT3 to promote Th17 cell generation by inducing the Aiolos expression and suppressing IL-2 expression (84). AhR also inhibits the activation of STAT1 and STAT5, which are inhibitory factors during Th17 cell differentiation (85). Meanwhile, AhR can regulate the expression of IL-17 by binding to the *Il17* gene locus (86). It has been certificated that IL-6, TGF-β1 promotes regulatory/non-pathogenic Th17 cell differentiation, whereas IL-23, IL-6, TGF-β3 induced pathogenic Th17 cell differentiation and IL-17 secretion (87–89). Interestingly, TGF-β1 and IL-6 induce the expression of AhR and c-Maf, transactivating IL-10 production in non-pathogenic Th17 cells. While in pathogenic Th17 cells, AhR expression was significantly downregulated (89). It seems that AhR is involved in the differentiation of pathogenic/non-pathogenic Th17 cells, though the specific mechanism is not clarified.

### 3.4 AhR regulates tissue-resident memory T cells

CD8^+^ T_RM_ cells expressing the characteristic markers CD103, CD69, and CD49a have been identified in the lesion of vitiligo patients and mouse models (90–92). T_RM_ cells develop and persist in the skin, produce IFN-γ, perforins, and granzyme B, and destroy melanocytes, causing the recurrence of depigmentation in the same area after treatment stops (93). Targeting the IL-15, such as anti-CD122 antibodies may be a potentially effective treatment approach to prevent recurrence (94). Recent research indicated that AhR is involved in modulating the differentiation and function of CD8^+^ T_RM_ cells (95, 96). AhR facilitates the persistence of T_RM_ cells in the epidermis by regulating the downstream target Blimp1, which contributes to tissue residency (95–97). Dean et al. found that AhR promotes T_RM_ cell differentiation while suppressing T_CM_ cells in intestinal tissue (96). Besides, AhR also regulates CD4^+^ T_RM_ cell differentiation and function, which may conduce to immune protection, and tissue remodeling in vitiligo (98).

### 3.5 AhR and other T cells

AhR signaling also regulates other T cell responses such as Th22 and Th9 cells. Th22 cells are a kind of CD4^+^ T cell subset that produces IL-22 in the absence of IL-17, showing a dual role in mucosal defense, tissue repair as well as inflammation and pathological change (99). Previous studies have shown that IL-22 was elevated in vitiligo and related to disease extent and activity (100, 101). AhR is the critical transcription factor for Th22 cell development (99). AhR activation enhanced IL-22 release in CD4^+^ T cells of vitiligo patients (102). AhR is also essential for Th9 cell development. Takami et al. indicated that exposure to AhR ligands could increase BATF expression and promote the differentiation of Th9 cells (103). Kumar et al. demonstrated the increased frequency of Th9 cells and elevated levels of IL-9 in active vitiligo patients, which contribute to reducing the IFNγ-induced oxidative stress in melanocytes (104). Thus, AhR activation may protect epidermal melanocytes from IFN-γ-induced damage by elevating the levels of IL-9.

## 4 AhR promotes melanogenesis and repigmentation

**Figure 3:**
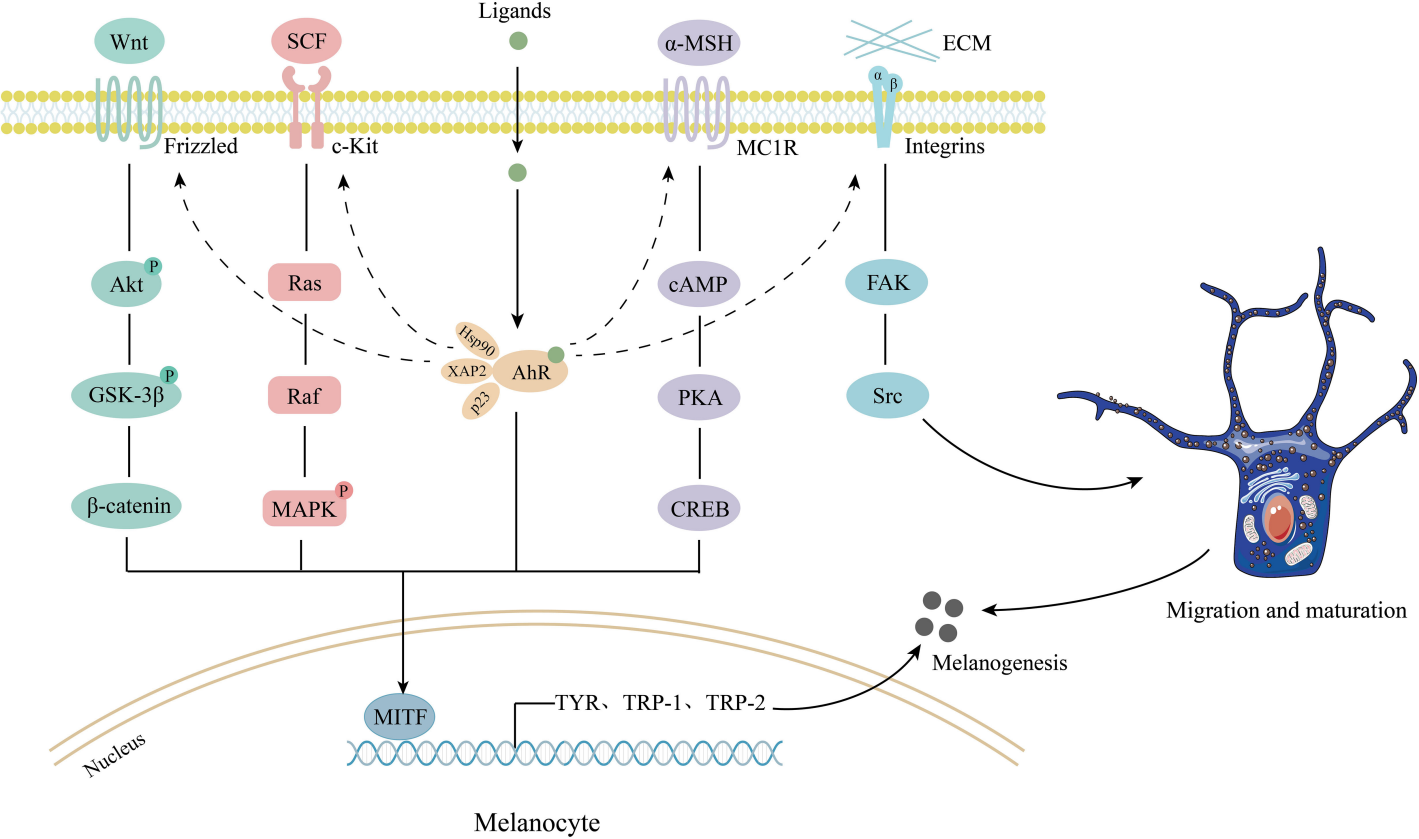
The possible mechanisms of AhR signaling in melanogenesis. AhR activation directly upregulates the expression of TYR, TRPs, and MITF. AhR crosstalks with SCF/c-Kit/MAPK, Wnt/β-catenin, α-MSH/cAMP/PKA, and FAK/Src pathways, mediating melanoblast migration, maturation, and melanin synthesis. Wnt, wingless/integrated; Akt, protein kinase B; P, phosphorylation; GSK-3β, glycogen synthase kinase 3β; SCF, stem cell factor; MAPK, mitogen-activated protein kinase; α-MSH, α–melanocyte-stimulating hormone; MC1R, melanocortin 1 receptor; c-AMP, cyclic adenosine monophosphate; PKA, c-AMP dependent protein kinase A; CREB, c-AMP response element binding protein; AhR, aromatic hydrocarbon receptor; Hsp90, heat shock protein 90; XAP-2, hepatitis B virus X-associated protein 2; p23, co-chaperone protein; FAK, focal adhesion kinase; MITF, microphthalmia-associated transcription factor; TYR, tyrosinase; TRP-1, tyrosinase-related protein 1; TRP-2, tyrosinase-related protein 2.

Apart from controlling disease progression through anti-oxidative stress and immunity modulation, AhR signaling pathway activation could result in the upregulation of melanogenesis-related gene expression, and melanin content production, thus promoting melanogenesis (**Figure 3**).

### 4.1 AhR directly upregulates TYR, TRP-1, TRP-2, and MITF expression

Melanin biosynthesis occurs in melanosomes of melanocytes, and its synthesis is regulated by the expression and activity of three melanogenic enzymes, tyrosinase (TYR), tyrosinase-related protein (TRP)-1, and TRP-2 (105). It has been documented that microphthalmia-associated transcription factor (MITF) is an important transcriptional regulator of pigmentation that can activate TYR, TRP-1, and TRP-2 expression (106).

Previous studies have shown that exposure to BA or TCDD can activate AhR and significantly increase TYR activity, melanin synthesis, as well as TRP-1, TRP-2, and MITF expression (38, 107). Luecke et al. suggested that the AhR signal may play a potential role in the melanogenesis of human melanocytes. They found that there were multiple XREs in the promoter regions, introns, and 3′ noncoding regions of TYR and TRPs, implicating they were the transcriptional targets of AhR (26). Studies on human populations also demonstrated that the skin hyperpigmentation rate is related to the blood level of AhR ligand polychlorinated biphenyls (108). Thus, AhR might be involved in melanogenesis and pigmentation by upregulating the TYR activity.

### 4.2 AhR regulates the SCF/c-Kit/MAPK pathway

Stem cell factor (SCF) and its receptor (c‐Kit) play vital roles in the survival, migration, proliferation, and differentiation of melanoblast (109). It has been confirmed that the expression of SCF, c‐Kit, and its downstream effector MITF significantly declined in vitiligo lesional and perilesional skin (110). Defects in SCF/c-Kit pathway may be related to melanocyte apoptosis in vitiligo. Furthermore, modulating SCF or c‐Kit expression, including narrow-band ultraviolet (UV) B radiation, psoralen, tacrolimus ointment, and geniposide, is the common treatment for vitiligo, which further indicates the involvement of abnormal SCF/c‐Kit signaling pathway in vitiligo (111, 112).

Jux et al. showed that UVB-induced TYR activity and melanocyte density are prominently diminished in AhR-deficient mice, which are associated with reduced SCF and c‐Kit expression in melanocytes (113). It is widely known that the promoter of *c-Kit* gene contains functional XREs which are addressed by AhR, suggesting that AhR signaling contributes to melanocyte homeostasis and differentiation by controlling SCF/c-Kit expression (114). Besides, AhR signaling also crosstalks with mitogen-activated protein kinase (MAPK) pathway, which is the downstream signaling of SCF/c-kit. Shi et al. revealed that PM2.5 significantly increases the phosphorylation level of MAPK proteins in an AhR-dependent manner and thereby promotes pigmentation (115). Thus, AhR signaling can upregulate the SCF/c-Kit/MAPK pathway and promote pigment synthesis.

### 4.3 AhR regulates the Wnt/β-catenin pathway

The Wnt signaling pathway also makes significant contributions to melanocyte development and melanogenesis. Wnt1 and Wnt3a facilitate the differentiation of neural crest cells into melanocytes (116). β-catenin binds with the lymphoid enhancer-binding factor 1 (LEF1) and synergistically upregulates MITF expression and promotes melanogenesis. Accumulating evidence has shown an alteration of the Wnt/β-catenin pathway in vitiligo lesions (117). Regazzetti et al. have demonstrated the suppression of the Wnt/β-catenin pathway in melanocytes and keratinocytes under oxidative stress. It was found that the expression of Wnt pathway components such as LEF1, CDH2, and CDH3 was downregulated in the lesional skin of vitiligo (118). In addition, it was found that the expression of Wnt pathway components such as LEF1, CDH2, and CDH3 was downregulated in the lesional skin of vitiligo (118). One study showed that narrow-band UVB could activate the Wnt/β-catenin pathway and strongly promote vitiligo repigmentation (119). Moreover, the Wnt pathway also has significant effects in regulating immunity and protecting melanocytes from oxidative stress, indicating that Wnt/β-catenin signaling is likely to be a valuable target to treat vitiligo (40).

The crosstalk between AhR and Wnt/β-catenin pathway has been studied. A previous study reported that β-catenin expression was significantly increased in melanocytes treated with AhR ligands (120). The β-catenin can physically interact with AhR in DNA-binding sites (121). Our study found that the activation of AhR-dependent AKT/GSK-3β/β-catenin pathway can further upregulate MITF transcription and enhance the expression of TYR, TRP-1, and TRP-2, ultimately promoting melanogenesis (122).

### 4.4 AhR is required for α-MSH-induced melanogenesis

α–melanocyte-stimulating hormone (α-MSH) is a key regulatory protein controlling melanocyte proliferation and melanin synthesis through cAMP/PKA/CREB/MITF, MAPK-ERK, and the Wnt signaling pathway (123–126). Melanocortin system defects have been discovered in vitiligo patients, including low α-MSH levels in serum, plasma, and lesional skin (127–130). Related synthetic analogue, such as Afamelanotide which can bind the melanocortin-1 receptor and stimulate melanogenesis, has been used as an adjuvant to increase therapeutic response to UVB phototherapy (131–133). The involvement of AhR in α-MSH-induced melanogenesis was reported. Bahraman’s study indicated that α-MSH increased melanin synthesis by upregulating the transcriptional level of AhR, CTNNB1, MITF, and TYR. Induction of α-MSH-stimulated melanogenesis may require the concomitant presence of AhR (121).

### 4.5 AhR mediates the proliferation, migration, and maturation of melanoblast

Repigmentation of vitiligo lesions hinges on melanoblast proliferation and migration from hair follicles to the epidermis. UV light therapy, including PUVA, NB-UVB, and excimer laser can stimulate melanoblast proliferation and upward migration to the epidermis, which promotes functional maturation of melanoblasts to melanin-producing melanocytes (134). AhR can regulate the early activation of focal adhesion kinase (FAK) and Src and mediate melanoblast maturation. Tomkiewicz et al. demonstrated that TCDD-mediated AhR activation resulted in the rapid activation of integrin clustering and FAK/Src, which had prominent effects on cell migration and integrin recycling (135). It suggested that AhR played a regulatory role in the migration of melanoblasts via the FAK signaling pathway. Moreover, AhR mediated the development and maturation of melanoblasts as well (136, 137). Hence, the AhR signaling pathway could promote melanin synthesis through multiple regulation mechanisms and is of great significance in repigmentation.

## 5 Discussion

Vitiligo is a chronic autoimmune skin disease characterized by melanocyte destruction and skin depigmentation. Due to the diversity and complexity of pathogenesis, there is no definitive safe and efficacious treatment for vitiligo. Current therapeutic strategies mainly include topical and systemic medications, phototherapy, and surgical grafting (138, 139). However, they are not universally effective in all patients and provide only short-term benefits with a high recurrence rate after discontinuing treatment. Thus, the search for more effective, targeted therapies is necessary. Current opinions suggest that the treatment of vitiligo requires a multimodal approach targeting three different aspects at the same time. The minimization of oxidative stress, immunomodulatory and immunosuppressant action, and regeneration of melanocytes may control the excessive oxidative damage and abnormal immune response so that prevent the progress of active disease as well as promote repigmentation, giving the best results in treating vitiligo.

Our review shows that AhR may be a potential target for vitiligo treatment. Current AhR agonists and antagonists studied in vitiligo are summarized in **Table 1**. In fact, narrow-band UVB, the most widely used therapy for vitiligo, probably induces repigmentation by activating AhR signaling (113). AhR can protect melanocytes from oxidate stress damage by activating the Nrf2-ARE pathway, which is already recognized as an attractive target for vitiligo treatment. More importantly, the activation of AhR signaling inhibits abnormal immune response and drives melanogenesis by upregulating the expression of melanogenic genes and transcription factors, which contribute to disease control and repigmentation. In addition, AhR also crosstalks with the JAK-STAT pathway, which is recognized as a key new therapeutic target for vitiligo.

**Table 1:** AhR agonists and antagonists studied in vitiligo.

Activity	Compounds	Reference
AhR Agonists	2,3,7,8-Tetrachlorodibenzo-p-dioxin (TCDD)	(26)
	6-Formylindolo [3,2-b] carbazole (FICZ)	(140)
	Flavonoids	(102, 122)
	Tarpinarof	(141)
	Tryptophan	(142)
	Kynurenine (Kyn)	(143)
	5-Hydroxyindole-3-acetic acid (5-HIAA)	(144)
AhR Antagonists	Cinnamaldehyde	(145)
	CH223191	(122)

However, there are still some limitations. Firstly, the antioxidant response mediated by the activation of AhR/Nrf2 pathway relies on the type of AhR ligand. Ketoconazole and cynaropicrin activate the AhR signaling pathway without ROS, while Dioxins, BaP, and other polycyclic aromatic hydrocarbons induce extremely high CYP1A1 expression and ROS production. Selecting appropriate ligands or inhibiting the activity of CYP1A1 may contribute to less oxidative damage. Secondly, the function of AhR is complex. Overactivation of AhR signaling has been detected in several cancer types, including melanoma and cutaneous squamous cell carcinoma. Topical treatment may be considered to reduce the risk of concurrent cancer complications. Finally, AhR promotes the differentiation of CD8^+^ T_RM_ cells in the skin, which may lead to recurrence after discontinuing treatment.

## 6 Conclusion

Our review summarizes recent topics on AhR and vitiligo. AhR can activate antioxidant pathways, inhibit abnormal immunity response, and upregulate melanogenesis gene, thereby protecting melanocytes from oxidative stress damage, controlling disease progression, and promoting lesion repigmentation. Further in-depth research and the development of related drugs may be beneficial for vitiligo.

## Author contributions

YL: Conceptualization, Investigation, Visualization, Writing – original draft. YZ: Funding acquisition, Investigation, Writing – review & editing. ZC: Investigation, Writing – review & editing. XT: Investigation, Writing – review & editing. XM: Investigation, Writing – review & editing. ZW: Conceptualization, Writing – review & editing.
